# Human Rights in the Least Developed Countries of Asia: An Index for Quantifying Sustainable Development Goal 3 (Good Health and Wellbeing)

**DOI:** 10.3390/ijerph18094747

**Published:** 2021-04-29

**Authors:** Karen G. Añaños Bedriñana, José Antonio Rodríguez Martín, Fanny T. Añaños

**Affiliations:** 1 Department of Constitutional Law, Institute of Peace and Conflicts Investigation (IPAZ), University of Granada, 18071 Granada, Spain; karengananos@ugr.es; 2 Department of Applied Economics, University of Granada, 18071 Granada, Spain; 3 Department of Pedagogy, Institute of Peace and Conflicts Investigation (IPAZ), University of Granada, 18071 Granada, Spain; fanntab@ugr.es

**Keywords:** health services research, human rights, least developed countries, maternal and child health, welfare

## Abstract

This paper aims to measure disparities among the variables associated with Sustainable Development Goal (SDG) 3 defined by the United Nations (UN) in the least developed countries (LDCs) of Asia. In the terms of the UN Conference on Trade and Development, LDCs are countries with profound economic and social inequalities. The indicator was constructed using a set of variables associated with SDG3: Good Health and Wellbeing. Applying Pena’s DP_2_ distance method to the most recent data available (2018) enables regional ordering of Asia’s LDCs based on the values of these variables. The index integrates socioeconomic variables that permit examination of the impact of each individual indicator to determine territorial disparities in terms of the partial indicators of SDG3. “Maternal education,” “Proportion of women who make their own informed decisions regarding sexual relations, contraceptive use, and reproductive health care,” and “Gender parity index in primary education” are the most important variables in explaining spatial disparities in good health and wellbeing in the LDCs of Asia.

## 1. Introduction

The 2030 Agenda for Sustainable Development, adopted by all United Nations (UN) Member States in 2015, provides a shared blueprint for peace and prosperity for people and the planet, now and into the future [1]. At its heart are the 17 Sustainable Development Goals (SDGs) [2]. These goals address the global challenges we face, including challenges related to poverty, inequality, climate change, environmental degradation, peace, and justice. The SDGs recognize that ending poverty and other deprivations must go hand-in-hand with strategies that improve health and education and reduce inequality, among other issues, all while tackling climate change [3].

Ensuring healthy lives and promoting wellbeing at all ages is essential to sustainable development. The world is currently facing a global health crisis unlike any other, and COVID-19 is increasing human suffering [4]. Before the pandemic, significant strides had been made in increasing life expectancy and reducing some of the common killers associated with child and maternal mortality. The Least Developed Countries (LDCs) have made substantial progress on child survival and maternal health since 2000, due among other factors to the improvement of maternal and child nutrition as well as the greater efficacy of vaccination and mother–child health care [5]. More effort is needed, however, to fully eradicate a wide range of diseases and address many different persistent and emerging health problems [6].

In contrast to the Millennium Development Goals (MDG), the SDGs are broader in scope and take a more holistic multisectoral approach to development [7]. While maintaining the MDGs’ health agenda, SDG3 embraces the growing challenge of non-communicable diseases and their risk factors. SDGs are interdependent; attaining targets in one goal contributes to attaining the targets in other goals [8]. Ill health is both a consequence and a cause of poverty [9]. Fullness, understood in terms of wellbeing, is related to the protection of health and of the environment [10].

The UN Development Programme highlighted huge disparities in countries’ abilities to cope with and recover from the COVID-19 crisis. Antimicrobial resistance also requires action [11]. The pandemic represents a watershed for health emergency preparedness, and for study and regional comparison of Asia’s poorest poor countries in terms of the variables of UNSDG3: Good Health and Wellbeing. Good health is essential to sustainable development, and the 2030 Agenda reflects the complexity and interconnectedness of the two [9].

In this study we use the term maternal education broadly, to encompass a greater number of years spent in education. We suggest that maternal education increases mothers’ access to human, cultural, and social capital and that mothers then use these forms of capital in a variety of ways to promote their children’s academic outcomes [1,2,11]. Maternal education is also a key factor in reducing child mortality [12]. Gakidou et al. [13] estimate that increases in maternal education may have accounted for the more than 50% of worldwide reduction in under-5 mortality between 1970 and 2009. But maternal education can also increase children’s health outcomes through a range of other factors, such as attitudes, knowledge, and women’s power [14]. Completing primary education results in greater chances of controlling various measures of personal illness (e.g., seeking treatment for one’s child’s cough/fever, receiving prenatal care in pregnancy, and receiving prenatal care in the first trimester) [15]. Education is also related to knowledge of contraceptive methods, more efficient contraceptive use, and the roles women play in society [16].

If current trends continue, with more than 50 countries falling short of the SDG target for child survival, some 60 million children under age 5 will die between 2017 and 2030—and half of them will be newborns [4]. If every country achieves the SDG target for child survival by 2030, the lives of an additional 10 million children under age 5 will be saved during the period 2017–2030—about half of them newborns. Inequities in child mortality remain large both across and within countries [6]. Substantial increase in education, especially of women, and reduction of the gender gap have significant implications, not only for health but also for women’s status and roles in society [17].

For millions of men, women, and children in the LDCs, development is an urgent human rights imperative [18]. The Istanbul Programme of Action contains many references to human rights, including the right to development, food, health, sexual and reproductive health, gender equality, and the empowerment of women [11]. Explanations of maternal mortality must include socioeconomic factors such as income and literacy. To lower morbidity levels, maternal morbidity and maternal mortality research must refocus to consider social factors such as literacy in the development of broad-based social programs [19], since the gains from improvements in female literacy reduce maternal morbidity and maternal mortality [20].

In Burkina Faso, research shows that mothers’ education significantly and positively affects children’s weight-for-height (which measures wasting, an indicator of the presence of malnutrition) [21]. In Sub-Saharan countries, it has been shown that mothers’ education level is an important determinant of child mortality [22], and studies confirm that children of more educated mothers have less risk of dying before their fifth birthday than do children born to mothers without formal education [23].

This study develops an indicator, known as Pena’s DP_2_ distance method [24,25,26,27,28,29,30,31,32,33,34,35,36,37,38], to measure fulfilment of the UN’s SDG3 in the LDCs of Asia. We use this indicator to analyze the disparities in 2018, taking SDG3 as the reference within the framework of the 2030 Agenda for Sustainable Development [3]. The indicator thus permits comparison among the LDCs of Asia in 2018, using as a reference the information contained in a set of social indicators detailed by the UN under SDG3. Comparing the degree to which each country has fulfilled SDG3 in countries at this date will enable us to confirm whether progress toward Good Health and Wellbeing has been more or less unequal to date in the LDCs of Asia. That is, our index contributes to the comparative analysis of each country in the context of the region and provides a methodology that approximates measures of SDG3.

Progress has been made toward the SDGs, but achievements have been uneven in the LCDs of Asia [3], one of the most impoverished areas of the planet [5]. Our index is significant in that it has not been applied previously to this group of countries in 2018, uses the latest data available, takes into account the criteria defined in the UN’s SDGs, and applies a multidimensional approach.

We chose this framework because of the detailed, more reliable statistical information contained in the report on the SDGs coordinated by the UN Statistics Division. These statistics are jointly compiled using data collected through the work of the UN Statistical Commission, which created the Inter-agency and Expert Group on SDG Indicators (IAEG-SDGs). Where information on SDG3 was not available for 2018, we used the nearest year as an alternative. Countries and UN agencies require reliable, high quality data to ensure that the world successfully meets the SDGs [39]. A robust follow-up and review mechanism for implementation of the 2030 Agenda for Sustainable Development requires a solid framework of indicators and statistical data to monitor progress, inform policy, and ensure accountability of all stakeholders. The global indicator framework was adopted by the General Assembly on 6 July 2017 and is contained in the Resolution adopted by the General Assembly on Work of the Statistical Commission pertaining to the 2030 Agenda for Sustainable Development [1,3].

The synthetic indicator also enables measurement of the impact of each variable individually in determining the outcomes and comparing them across countries to determine disparities in the variables associated with SDG3 for each LDC of Asia [28]. This method explores the relative impact of each variable by using the correction factor [18,40,41,42,43].

The LDCs are a list of developing countries that the UN ranks as showing the lowest indicators of socioeconomic development, with the lowest Human Development Index (HDI) ratings in the world [5]. The HDI compiles information on three dimensions: education, health, and living standards [28,44].

## 2. Methodology

There are different approaches to aggregating indicators to construct a composite index [36,45]. The methodology used in our study is based on the construction of a synthetic index as a function of a set of intermediate social variables that help to quantify some aspect of the concept to be synthesized, following the OECD methodology [46]—in our case fulfillment of UN SDG3. As to effect size, the larger the country the higher generally the values of the observable variables. Thus, to relativize the observed values of DP2, it is sufficient to express the variables as a function of the population or of the surface area, depending on whether their respective values increase with the population or with the surface area [22]. This modelling method enables us to analyze a large set of data through one synthetic indicator—the DP_2_ indicator. We can then compare countries using not just one indicator but a whole set of indicators synthesized into one index [42,43]. In contrast to the dissemination of information that may be derived from one-dimensional indicators [47,48], synthetic indicators (applied in our study) integrate all information on the variables related to level of achievement of SDG3 into the synthetic indicator DP_2_.

More specifically, we used the following partial indicators of UN SDG3, based on the most recent data and variables available, taking the variables of the UN’s SDGs as a reference (for definitions, see Appendix A [3,4,11]): (a) Primary completion rate for girls and boys; (b) Proportion of women aged 15–49 years who make their own informed decisions regarding sexual relations, contraceptive use, and reproductive health care; (c) Gender parity index in primary education; (d) Attended by skilled health personnel, percentage; (e) Children 1 year old immunized against measles, percentage; (f) Infant mortality rate (0–1 year) per 1000 live births; (g) Literacy rate of 15–24 year-olds, women, and men; (h) Maternal mortality ratio per 100,000 live births.

As mentioned above, this study develops a synthetic indicator with a special focus, the DP_2_ distance method. This method is designed to make comparisons among regions and has been used in a number of studies. Currently, DP_2_ is widely used by scientists around the world to measure welfare, quality of life, and other similar concepts, such as poverty, at regional, national, and SDG levels [17,18,22,24,26,27,29,30,31,32,33,34,35,36,37,38,40,41,42,43,47,48,49,50,51,52,53,54,55,56,57,58,59,60,61,62,63,64]. Our article uses this method to measure fulfilment of SDG3 on Good Health and Wellbeing, as defined by the UN in the LDCs of Asia, enabling comparison of nine countries using SDG3 as a reference. To achieve our goal, our article is organized around the methodology of the DP_2_ indicator, based on its main mathematical properties and advantages. This technique verifies all of the mathematical properties required of a good synthetic social indicator and enables estimation of regional disparities [27,29,30,32,35,37,40,47,48,49,50,51,52]. It also guarantees that the weight of the partial indicators is determined in a non-arbitrary manner [36].

The main problems to be solved when measuring social welfare or any other multidimensional concept through the social indicator approach are selecting the areas into which the concept may be broken down, choosing which simple or partial indicators are appropriate statistical measures for measuring each area, and aggregating the simple indicators through a suitable synthetic indicator [2,25]. As mentioned above, we chose the multidimensional DP_2_ method because it overcomes the main limitations of a synthetic indicator [26,27,28,30], specifically, the issues of aggregation of variables expressed in different measurements, lack of neutrality in weighting of the partial indicators, and redundancy of information [48,50].

The DP_2_ method is also appropriate when applying the social indicators approach [30,34], the approach adopted in this study. Moreover, it has been proven appropriate for resolving issues that other methods fail to treat adequately [35,48,49,50,51]. Thus, to analyze the data, we have used the DP_2_ of the Pena distance method, while explaining this methodology in the study. This measurement method verifies a set of properties such that the weight of the partial indicators is determined in a non-arbitrary manner, thus permitting interpretation of the results from an economic standpoint [35,50,52,53,54]. Further, since this method produces a cardinal measurement of distance, comparisons can be made in time and space [17,28,29,30].

To evaluate SDG3, it is necessary to use various social indicators simultaneously [25]. In this multidimensional evaluation, defining an appropriate aggregation method to combine multidimensional variables in an overall index is extremely important [40]. Since DP_2_ is a numerical value, it also verifies this property [18,30,34,35]. Since the goal is to measure the level of Good Health and Wellbeing in different countries to establish comparisons, the DP_2_ synthetic indicator includes the disparities in SDG3. With a view to the upcoming 2030 timeframe for the final assessment of progress towards the SDGs, this study investigates achievement of SDG3 in nine countries in the LCDs of Asia in the year 2018 based on two approaches. In the first phase, it applies the Pena distance method to compare each country individually, while also determining each country’s situation relative to the context of LCDs in Asia.

As to selection of the countries, this article proposes to measure disparities between indicators of SDG3, applied to a group of countries especially affected by poverty—the LDCs which, in the terminology of the United Nations Conference on Trade and Development (UNCTAD), are the poorest of the poor. These countries have serious deficiencies and economic and social inequalities [5,11], within a territory that gives them homogeneity—the continent of Asia—and that need special support from the international community [5]. LDCs are generally considered as requiring the highest level of attention from the international community, more than other countries, as they are not able to emerge from their situation poverty [28,29,30,31,32,33,34,35,36,37,38,39,40,41,42,43,44]. The UN sends a strong signal to the development partners of these countries and points to the need for special international support measures and concessions in their favor [5,11]. The countries analyzed are Bhutan, Cambodia, Myanmar, Bangladesh, Lao People’s Democratic Republic, Timor-Leste, Yemen, and Afghanistan.

The DP_2_ distance method gives us a global synthetic indicator with a holistic view of the situation of the LCDs of Asia relative to Good Health and Wellbeing. We can then examine the impact of each SDG3 variable on the final outcome of the synthetic indicator and the discriminatory power or relevance of each variable. A higher DP_2_ value reflects a greater degree of achievement of SDG3, as it means greater distance from the theoretically “least desirable” situation [29,30] (as we shall explain later). The index enables classification of the countries and examination of the impact of each individual indicator to determine country disparities in fulfilment of SDG3. Among other objectives, this article thus seeks to obtain a robust synthetic measure of SDG3 levels based on the most recent information available and to analyze intercountry disparities, exploring the impact of each simple indicator in determining these disparities. Conclusions can be obtained concerning which issues prove most relevant when explaining spatial disparities in levels of Good Health and Wellbeing. That is, a simple indicator with a high discriminant capacity will significantly impact the results [30].

The distances are estimated by considering two countries, taking one as a reference point, and the resulting value is divided by the standard deviation [36]. Thus, variables are expressed in abstract units beyond the initial units of the single indicators, allowing the single indicators to be aggregated into a composite index [51,52].

The DP_2_ fulfils the following properties to provide a good measurement or estimation of the object to be measured. The DP_2_ verifies a set of “properties required for a good synthetic indicator” [25,27,29,30,31,33,34,40,48,51,53,54,55] and, as argued by Zarzosa and Somarriba [30], turns out to be adequate since it uses ad-hoc measurements specifically designed to measure distance between different situations: existence and determination, monotony, uniqueness, grade-one homogeneity, transitivity, and neutrality. In addition to these properties, we again stress that the indicator solves a large number of problems, such as aggregation of variables expressed in different measures, arbitrary weights, and duplicity of information [22,26,30,31,41,43,47,49,50,54,55,56,57].

The DP_2_ indicator is a cardinal measure that enables comparisons between units across space and/or time and in which the weights allow for a clearer interpretation and inter-spatial comparisons [35,58]. In our study, the weights of each variable are determined from an initial situation that is determined from the absolute values of the coefficients of linear correlation between the values of the indicators and the synthetic indicator [2,34,56,58]. This indicator thus permits comparison of countries in 2018, using as a baseline reference the information contained in a set of social indicators established by the UN under SDG3 (Table 1).

### Description of the Statistical Model

It is important to define an appropriate aggregation method for synthesizing the information to combine variables in an overall synthetic index, as a cardinal measurement, permitting comparison among different countries [17]. As mentioned above, the methodological approaches used in this study are based on construction of a synthetic indicator that follows Pena’s method (DP_2_) [34,35,36,43,54]. The DP_2_ provides an ideal solution to the problems involved in devising a synthetic indicator [17,29,32,35,59].

The input order of the variables governing the relative weight of each variable is determined by an algorithm that reaches convergence when the indicator fulfils a number of desirable properties [18,30,31,32,34,38,48,50,59].

As mentioned above, the DP_2_ measures the distance between the issue studied in each country and a fictitious base reference. Here, we take as reference a theoretical country that obtains the worst values for the variables studied. The base reference is assigned a value of zero in the synthetic indicator of SDG3. The DP_2_ indicator calculates the distance of each country from that theoretical country of reference [18,28,29,30,32,33,48,54,55,56,60,61].

The base reference thus represents the results for an imaginary country that reflects the worst possible scenario for all variables [50] and would therefore be assigned a value of zero in the synthetic SDG3 indicator. By dividing it by a standard deviation, we solve the problem of heterogeneity of the units in which the variables are measured by expressing all partial indicators in abstract units [31,34,42,62,63].

The DP_2_ distance from country *r* is defined as follows [24,25,32,41,55]:

*DP*_2_=∑ {(*d_i_*/*σ_i_*)(1−*R*^2^*_i_*_,_*_i_*_−__1__,…,__1_)} (1)

where *R*^2^_1_ = 0; *di* = |*x_ji_* − x*_i_| is the base reference X* = (X*_1_, X*_2_,....X*_n_), which coincides with the minimum vector; *n* is the number of variables; *x_ri_* is the value of variable *i* in country *r*; *σ_i_* is the standard deviation of variable *I*; *R*^2^*_i_*_,*i*−1,…,1_ is the determination coefficient in regression *X_i_* over *X_i−_*_1_, *X_i−_*_2_,…,*X*_1_, which is already included; and (1 − *R^2^_i_*_,*i−*1,…,1_) is a “correction factor” that avoids redundancy, since it discards from each new variable the information already included in previous variables, eliminating the portion of information already explained [24,25,33,34,61].

More specifically, the correction factor prevents duplication of information by excluding information contained in the variables that precede each variable considered [18,28,30,33,40,51,52,56,64].

In addition, the variables are arranged in descending order of correlation with this indicator. The differences in the *i-*th variable between a country and the reference base are thus weighted by the percentage of new information not included in the other variables [29,35,60,61].

As indicated above, the aim of this study is to construct a synthetic indicator of SDG3 in the LDCs of Asia and analyze existing disparities, using the UN SDGs for Good Health and Wellbeing as a reference (Table 1). The year of analysis is 2018, the latest year for which data are available. For variables for which 2018 information was not available, we took the nearest year as an alternative.

Finally, to ensure that the properties of the synthetic indicator are fulfilled, we note that variables bearing a negative relation to SDG3 are reflected in the matrix of observations X with a negative sign (−), while variables bearing a positive relationship take a positive sign (+). That is, to guarantee fulfilment of the properties of the synthetic indicator [34,35,38], we multiply specific variables whose increase implies a worsening of Good Health and Wellbeing by −1 (Table 2). An increase in the value of any variable might therefore mean an improvement in SDG3. For example, the variable Gender parity index in primary education has a positive sign because it has a positive relationship to Good Health and Wellbeing. The variable maternal mortality ratio per 100,000 live births, in contrast, has a negative sign because it has a negative relationship to Good Health and Wellbeing (i.e., increases would be associated with reductions in Good Health and Wellbeing).

## 3. Results

### 3.1. Results of the Indicator—Country Classification

To obtain the final result by country, we took as a reference the worst theoretical situation of a country [30], that is, the minimum values in the set of SDG3 variables considered [28,49]. Higher values for the distance measured thus mean that a country is farther from this minimum value for Good Health and Wellbeing.

We could also have taken as a reference a theoretical country that registered the maximum values in the set of variables studies [34,35,40,55,60].

The systemic indicator shows that Bhutan (with a distance from the baseline of 8.23) obtained the best results on SDG3: Good Health and Wellbeing. Nepal and Cambodia follow, with relatively high values in most of the SDG3 variables and values above the average of the set of countries included in the 2018 ranking (Table 1).

Bhutan’s primary school completion rate is estimated at 89% but is higher for females than for males. This figure is also much higher in the urban than in the rural areas, however, indicating that Bhutan still needs attention and encouragement even though it remains firmly on track to achieve SDG3. The targets that have been achieved already include lowering the percentage of underweight young children and increasing gross enrolment of children in primary schooling.

Afghanistan, Yemen, Timor-Leste, and Lao People’s Democratic Republic, in contrast, were among the countries with the worst theoretical situations in progress toward SDG3 (Table 1), progress that has been uneven. Given their low values in most of the variables of Good Health and Wellbeing analyzed, especially variables associated with girls’ education and women’s universal access to sexual and reproductive health, these results are not surprising.

The 2018 results indicate a high maximum distance between countries—that is, between the maximum and minimum values for disparities in women’s education and child health in the LDCs of Asia surveyed. Based on the foregoing, the maximum intercountry distance—the distance between the maximum and minimum values obtained—was quite high. Specifically, the difference between the value assigned the first country, Bhutan, and the last, Afghanistan, was 8.06 (Table 1).

Bhutan, and to a lesser extent Nepal, achieve far more positive results than other countries in the LDCs of Asia for the indicators on SDG3 included in our study (Table 1). Yemen and Afghanistan, in contrast, are among the countries with the worst theoretical scenarios, showing a distance from the baseline of 3.29 and 0.17, respectively. In Yemen’s crisis, the world’s greatest humanitarian and development disaster is being exacerbated by the unmitigated spread of Coronavirus (COVID-19). Yemen’s case fatality is four to six times higher than the global average. In a country of 30 million, nearly 80% of Yemenis needs assistance [11].

Finally, Afghanistan has the lowest enrolment ratios in primary education rate for girls and boys and the lowest gender parity index in primary education among the LDCs of Asia. Afghanistan accounts for over 9% of the population total of LDCs of Asia. Its below-average values relatively close to the minimum reference threshold (Table 1) indicate that a large proportion of the country’s population is at a severe disadvantage for being on track to achieve SDG3. Accessing health care and reducing pregnancy and newborn deaths are a key part of SDG3 [65], but Brown University [66] reported very limited access to health facilities in Afghanistan. This finding is not surprising, given that UN statistics show that Afghanistan still obtains very negative results on basic variables for wellbeing and health compared to the higher values of the other countries in the region.

Although it is highly likely that none of the countries in the region will achieve SDG3 by 2030, Bhutan, and to a lesser extent Nepal and Cambodia, will achieve far more positive results than others for the indicators on the goal “Ensure healthy lives and promote well-being for all at all ages” considered in our study. These countries have made the greatest gains against several of the leading causes of death and disease in the region. In general, the DP_2_ classification of SDG3 for LDCs of Asia does not coincide with the HDI classification of countries with low human development. In contrast to these data, the HDI’s classification of the LDCs of Asia ranked Timor-Leste last (in contrast to 7th position in the DP_2_ classification), with relatively low values in most of the SDG3 variables, whereas Bangladesh (5th position) (Table 1) had an average level of development in both cases [11].

Apart from Afghanistan, Yemen, and Timor-Leste, the disparities in progress toward SDG3 relative to the variables analyzed were not very high in the year studied. We must thus pay special attention to these variables through specific aid programs to improve Good Health and Wellbeing in the coming years in these three countries to help them progress in the 2030 timeframe and beyond. Finally, we reiterate our intention to continue working in this line of research within the framework of the Post-2015 Agenda of SDGs.

### 3.2. Ordering of the Partial Indicators

The results of our study reaffirm the advantages of the method used, which eliminates duplicity of information among the variables considered. Table 2 presents the partial indicators in order of their correction factors. We calculated the correction factor of the variables associated with SDG**3**, which indicate the relative importance of each partial indicator included in the final indicator of SDG3: Good Health and Wellbeing in Asia’s LDCs. The first variable in entry order is “Primary completion rate for girls and boys,” which contributes the most new and useful information for estimating the final indicator of SDG3. This value means that 0% of the variable’s variance was explained by a preceding variable and thus that 100% of the new information was incorporated [31,36,40,54,64].

The next-highest partial indicator in the correction factor, or specific weight in the SDG3 indicator, is “Gender parity index in primary education,” a variable associated with gender equality in the education of young women and girls in these countries. This partial indicator is followed by the third-ranked “Proportion of women aged 15–49 years who make their own informed decisions regarding sexual relations, contraceptive use and reproductive health care.” These three variables—all connected to women’s education, gender equality, and reproductive health—correlate highly with achieving a higher level of UN SDG3 in the countries analyzed.

Attending to the order of the variables (Table 2) in general, we ascertain that the variables associated with gender equality in education and universal access to sexual and reproductive health services—including those for family planning, information, and education—had greater influence and significance in determining the level of SDG3: Good Health and Wellbeing in the LDCs of Asia in 2018. These areas thus constitute a key field of action in which to improve health and wellbeing in these countries in the coming decades within the framework of Agenda 2030 for Sustainable Development and beyond.

Finally, it should be stressed that the selection criterion DP_2_ only eliminates a variable completely when it does not add any new information to the measurement of SDG3: Good Health and Wellbeing. We therefore keep variables 6 to 8, probably because their information is included in other variables considered in this study.

### 3.3. Discriminating Power of the Variables

Finally, we estimated the power of the information from each of the variables considered. These values are displayed in the column “Ivanovic-Pena Overall Information Coefficient” (IC) [26,41,59,67,68]. The coefficient indicates the quantity of information the variable provides [30] and contains a high volume of new information on level of SDG3 achieved. Further, the greater the quantity of information contributed by an indicator not contained in the overall information of the indicators already incorporated into the synthetic indicator, the better the partial indicator [26,27,28,29,30].

If a variable is constant throughout the set of countries, it will have zero discriminating power (IC = 0) and its information will not be relevant in evaluating relative levels of Good Health and Wellbeing among the LDCs of Asia. If, in contrast, a variable discriminates completely (IC = 2), it provides very important information [68].

In other words, the less importance a variable acquires when the set of values for the variables is equal to and different from zero, the greater its relevance when all but one is null [26,28]. That is, obtaining a null value in this indicator does not mean that the variable does not contribute information but rather that its information has already been incorporated in the previous set of variables [40].

Thus, the variables that obtain higher IC values (Table 3)—that is, the variables that contribute the most information (i.e., the most discriminating indicators) to the SDG3—are, in order (as indicated by their position in Table 2 above) and according to the correction factor: 2. Gender parity index in primary education; 3. Proportion of women aged 15–49 years who make their own informed decisions regarding sexual relations, contraceptive use and reproductive health care; and 1. Primary completion rate for girls and boys (Table 3).

These three variables showed the greatest differences in values among the LDCs of Asia in 2018. Again, these are variables closely linked to gender equality in education and women’s access to, autonomy in, and information on issues related to reproductive health. We observe striking differences in the value of the variables in areas that play a key role in achieving SDG3 across countries. These areas also include variables directly related to child health: “Infant mortality rate (0–1 year) per 1000 live births.” With the exception of these variables, the variables do not show very unequal values among the countries in the region (Table 2 and Table 3). In fact, we find three minimally informative variables with almost no power of discrimination: “Maternal mortality per 100,000 live births” “Attended by skilled health personnel, percentage;” and “Children 1 year old immunized against measles, percentage.”

## 4. Discussion

The main contribution of this study was to provide a method to measure current progress toward SDGs in countries among the LDCs of Asia by building a global synthetic indicator for fulfilment of UN SDG3. This indicator enables establishment of comparisons among different territories at a given time. In contrast to the information that can be derived from one-dimensional indicators, synthetic indicators (which we apply in this study) integrate all information on the variables related to a society’s level of SDG3 into the synthetic indicator DP_2_ [41]. The variables are arranged in descending order according to their correlation with this indicator [40]. The indicator was constructed using a set of variables associated with SDG3: Good Health and Wellbeing, as defined by the UN. The indicator permits comparison of these partial indicators across countries during the second stage of the program to achieve SDG3: Good Health and Wellbeing among the LDCs of Asia by the target date of 2030. This agenda builds on the MDGs for 2015. To perform this measurement, we evaluated the regional value disparities between variables associated with comparative degree of fulfillment of these eight indicators of SDG3 in the nine LDCs of Asia.

To achieve this goal, we used the Pena Method DP_2_ based on the values of a number of variables for which consistent statistical information was available from the UN. This method overcomes limitations in the calculation of other indicators by excluding information contained in the variables that precede each of the variables considered, aggregating variables expressed in different measures and adjusting for arbitrary weights [25,26,28,30,43,62]. Thus, the method DP_2_ allows for the aggregation of variables expressed in different measures, avoiding arbitrary weights and duplication of information.

The results have implications for international development aid strategies in the context of Agenda 2030, which should aim to reduce territorial disparities in Good Health and Wellbeing in the LDCs of Asia in the near future, given their multiple objectives in the Agenda 2030 timeframe of prioritizing certain milestones. These strategies should target reducing territorial inequalities among countries of region in order to achieve better health and wellbeing more successfully, through special intervention programs in the lowest-ranked countries in our classification (Table 1).

Based on the most discriminant variables, we identify the most relevant issues and those with the greatest impact. These variables contribute to a greater extent to the countries’ progress toward reaching SDG3 in the coming years. Although countries among the LDCs of Asia are generally characterized as hopelessly backward economically, especially affected by poverty and, in the terms of UNCTAD, the poorest of the poor, we observe differences in their values for the SDG3 variables.

The need to make a greater effort to achieve SDG3 for 2030 is clear [3], especially in the LDCs of Asia. The goal is to reduce the global maternal mortality ratio to less than 70 per 100,000 live births by 2030. The results also have implications for the UN’s development aid strategies, which should aim to reduce territorial disparities in Good Health and Wellbeing and child health in the LDCs of Asia, in order ultimately to achieve better results for the variables considered in all countries in the region soon.

The values of the DP_2_ indicator demonstrate the existence of territorial disparities across LCDs of Asia with regard to SDG3 in 2018. The evidence shows that disparities in child survival and in SDG3 exist across regions and countries. Many lives can be saved by closing the gaps between countries [2,52]. The values of the DP_2_ indicator reveal strong polarization and disparity in the levels at which countries in the LDCs of Asia were meeting SDG3: Good Health and Wellbeing in 2018 (Table 1).

Our analysis shows significant differences in SDG3 across countries in the value of the significant variables “Ensure healthy lives” and “Promote well-being for all at all ages” (Table 3). The progress of Asia’s LDCs toward fulfilling SDG3: Good Health and Wellbeing is very uneven in key areas of gender equality and education of boys and girls. Levels are especially low for the variables “Primary completion rate” and “Gender parity index in primary education,” but also for variables associated with women’s universal access to sexual and reproductive healthcare, specifically “Proportion of women aged 15–49 years who make their own informed decisions regarding sexual relations, contraceptive use and reproductive health care.”

It is therefore essential for the international community to renew and increase its efforts through strategies and national programs to enable these countries to progress toward fulfilling SDG3 more quickly. These goals are crucial in helping to save the lives of millions of people in the poorest countries on the continent [3] in the near future, populations also powerfully affected by the COVID-19 health crisis [4].

For 2030, we must pay special attention to improving the situation in Asia’s LDCs. As our results show, doing so requires a quantified, regularly monitored, multidimensional focus on the intermediate objectives of the UN’s SDG3, with its fundamental elements such as gender equality, universal education (an important factor for improving maternal health and child health ages 3–5) and empowering all women and girls by guaranteeing information and universal access to sexual health and reproductive services.

All of these actions will unquestionably increase general wellbeing in this region, which has such great need for attention from international organizations and from the rest of the world. Although countries will probably need more resources to achieve SDG3, other constraints in the broader health system must also be addressed. In line with the analysis and results of other studies of these countries, these constraints include inefficient allocation of resources across interventions and populations, weak governance systems, human resource shortages, and drug shortages [69,70].

International organisms and international donors should provide sufficient technical and financial resources dedicated specifically to the LDCs of Asia’s SDG program [70], with special attention to the countries ranked lowest in our synthetic indicator for SDG3 (Table 1).

The health and wellbeing incorporated into SDG3 are influenced by multiple variables that are interrelated amongst themselves. In recent decades, significant advances have been achieved in increased life expectancy and reduction of child and maternal mortality in many countries [6]. Progress has been uneven across countries and regions, however, and especially delayed in many LDCs of Asia, which are falling way behind in achieving the health-related SDGs. It is essential to track whether these goals will be fulfilled by 2030, and much greater effort is needed to control a wide range of persistent and emerging illnesses and health problems.

Finally, we would like to emphasize that our ultimate goal is to use the latest data available to provide new information that sheds a little more light on the progress made towards SDG3 in the LDCs of Asia and on the factors most relevant to fostering Good Health and Wellbeing of the population in the region. We reiterate our desire that this study constitute a significant contribution to attempts by other researchers who share our goal to measure and improve the quality of life and good health—in this case, of the LDCs of Asia, prioritizing specific intermediate goals in order to keep advancing toward achievement of SDG3 within the 2030 timeframe. We greatly welcome other new initiatives on this topic. Our aim is to further these countries’ progress in achieving SDG3.

## 5. Conclusions

This study has developed a regional measurement of the LDCs of Asia. From this measurement, we conclude that the most significant differences between these countries are linked to fundamental variables of education of girls and associated with women’s universal access to sexual and reproductive healthcare and reproductive rights. We believe that the most significant gaps in progress toward SDG3: Good Health and Wellbeing among the LDCs of Asia correlate with variables of gender equality, maternal education, and universal access to information and services for women’s sexual and reproductive health, especially for the youngest and adolescents. Maternal education is a salient marker of advantage, not only for mothers themselves but also for their children [71].

All of the variables analyzed contribute relevant information to determining achievement level of SDG3 in the LDCs of Asia. The variables whose values differ most among countries are “Gender parity index in primary education;” “Proportion of women aged 15–49 years who make their own informed decisions regarding sexual relations, contraceptive use and reproductive health care;” and “Gender parity index in primary education.” These variables’ IC Ivanovic-Pena values are greater than those of the other variables. We find fewer territorial differences in the values of the variables associated with “Maternal mortality ratio per 100,000 live births.”

The target “Ensure universal access to sexual and reproductive healthcare services, including for family planning, information and education” and the integration of reproductive health into national strategies and programs will be integral to achieving SDG3 in the LDCs of Asia. Priority must be given to interventions to address the variables that have greater power to explain the differences in the values between countries relative primarily to the variable, “Gender parity index in primary education.” In this regard, and bearing in mind the value of the indicator obtained in this study, it is necessary to increase women’s participation to ensure universal access to sexual and reproductive healthcare services and to education, especially participation of the youngest. Women must also participate in initiatives aimed at the good health and wellbeing in these countries, requiring special attention to those ranked lowest according to the classification of our indicator (Table 1).

Based on the values of the variables associated with UN SDG3 analyzed here, the DP_2_ method shows high regional rates of difference in the quantitative synthetic indicator for Good Health and Wellbeing in the LDCs of Asia in 2018. More specifically, the values of the final indicator reveal a significant gap between the two highest-ranked countries (Bhutan, with a relatively small population in the region, the LDC closest to fulfilling SDG3 in 2018), Nepal, and the other LDCs.

Afghanistan, the country ranked last and in the theoretically least-desirable position, received the lowest values in the set of SDG3 variables studied. Between these extremes, the other countries are ranked as follows: Nepal, Cambodia, Myanmar, Bangladesh (with the highest population among these countries), Lao People’s Democratic Republic, Timor-Leste, and Yemen. By way of comparison, the first positions in this ranking of LDCs of Asia for fulfillment of UN SDG3 in 2018 do not agree with those in other studies of the region that used variables of MDGs. We highlight the finding that Myanmar now fallen 4 positions to 4th place is ranked in 2018 [28]. The order of the countries in the last positions is more consistent with that in other studies [28,50].

These results show the need for special aid programs that target healthcare for the countries ranked lowest, especially Afghanistan, Yemen (one of the largest countries geographically in the region), and Timor Leste, to enable them to come closer to fulfilling SDG3 in the next ten-year period. These countries are currently extremely far from achieving this basic goal for the wellbeing of their population. In general, the DP_2_ classification for these countries differs from that made by the HDI for countries with low human development in 2018.

According to the correction factors, the variables included in Target 2 (“Achieve universal primary education”) and Target 3 (“Achieve gender equality and empower women”) seemed to show the highest correlation to SDG3 in the LDCs of Asia in 2018. These targets have greater relative weight in determining the SDG3 indicator than do the other variables analyzed.

These variables can thus be considered as key elements for improving the value of the indicator in countries in the future by prioritizing specific intermediate goals in the following order: “Primary completion rate for girls and boys;” and “Proportion of women aged 15–49 years who make their own informed decisions regarding sexual relations, contraceptive use and reproductive health care.” These variables should receive more attention in the aid programs of international organizations, especially those of the UN, for whom our study was performed. The UN must step up efforts to further development in countries of the LDCs of Asia that show the lowest values in the variables analyzed here, as such efforts would undoubtedly result in greater Good Health and Wellbeing. The efforts should concentrate on the countries ranked lowest in our classification, targeting people highly vulnerable to poor health, lacking access to primary education, and marginalized on the grounds of gender. Among these groups, young women and reproductive health care require special emphasis. The fundamental causes of these gaps must also be addressed globally after 2030. In light of our results, further efforts are clearly needed to improve progress toward SDG3 if the greatest number of LDCs of Asia are to achieve—or come as close as possible to achieving—SDG3 by the year 2030.

These results will increase in significance when we obtain results from applying this technique and use of these variables to both LDCs in other regions globally and the LDCs of Asia in the coming years. Such data will enable us to capture any differences that may emerge.

## Figures and Tables

**Table 1:** Synthetic indicator of Sustainable Development Goal 3 (SDG3): Good Health and Wellbeing of the least developed countries (LDCs) of Asia for 2018. Countries in order of relative Pena Distance Method Indicator.

Classification	Country	DP_2_ Indicator
1	Bhutan	8.23
2	Nepal	7.84
3	Cambodia	7.74
4	Myanmar	7.48
5	Bangladesh	7.01
6	Lao People’s Democratic Republic	6.95
7	Timor-Leste	4.89
8	Yemen	3.29
9	Afghanistan *	0.17

* Afghanistan is the only country for which the original data are from the year 2017. Source: The authors, based on UN (2019) [3] and UNDP (2019, 2020) [4,11].

**Table 2:** Order of partial indicators of SDG3 according to correction factor and sign of variables’ relationship to increase in Good Health and Wellbeing 2018.

Position	Partial Indicators	Correction Factor
1	Primary completion rate for girls and boys (positive sign +)	1.00
2	Gender parity index in primary education (positive sign +)	0.44
3	Proportion of women aged 15–49 years who make their own informed decisions regarding sexual relations, contraceptive use and reproductive health care (positive sign +)	0.40
4	Attended by skilled health personnel, percentage (positive sign +)	0.27
5	Children 1 year old immunized against measles, percentage (positive sign +)	0.20
6	Infant mortality rate (0–1 year) per 1000 live births (negative sign −)	0.15
7	Literacy rate of 15–24 year-olds, women and men (positive sign +)	0.10
8	Maternal mortality ratio per 100,000 live births (negative sign −)	0.06

Source: The authors, based on UN (2019) [3] and UNDP (2019, 2020) [4,11].

**Table 3:** Amount of information on the variables on SDG3: Good Health and Wellbeing in LDCs of Asia, 2018.

Position	Partial Indicators	IC
1	Gender parity index in primary education	0.59
2	Proportion of women aged 15–49 years who make their own informed decisions regarding sexual relations, contraceptive use and reproductive health care	0.53
3	Primary completion rate for girls and boys	0.42
4	Infant mortality rate (0–1 year) per 1000 live births	0.26
5	Literacy rate of 15–24 year-olds, women and men	0.18
6	Maternal mortality ratio per 100,000 live births	0.10
7	Attended by skilled health personnel, percentage	0.09
8	Children 1 year old immunized against measles, percentage	0.04

Source: The authors, based on UN (2019) [3] and UNDP (2019, 2020) [4,11].

## Data Availability

The data presented in this study are available on request from the corresponding author.

## Appendix A

**Table A1:** Detailed description of proposed variables [1,3,4,7,11].

	Variable	Definition	Target
Variable 1	Gender parity index in primary education.	The ratio of girls to boys in primary education, or Gender Parity Index, is the ratio between the Gross Enrolment Ratio (GER) of girls and that of boys, for each level of education. Gender Parity Index is another term used.	Promote gender equality and empower women.
Variable 2	Proportion of women aged 15–49 years who make their own informed decisions regarding sexual.	Women’s and girls’ autonomy in decision making over consensual sexual relations, contraceptive use and access to sexual and reproductive health services is key to their empowerment and the full exercise of their reproductive rights.	Achieve gender equality and empower all women and girls
Variable 3	Primary completion rate for girls and boys.	Primary completion is measured by the Gross Intake Ratio, which is the total number of new entrants who reach the last grade of primary education, regardless of age, expressed as percentage of the total population of the theoretical entrance age to the last grade of primary education.	Ensure inclusive and equitable quality education and promote lifelong learning opportunities for all.
Variable 4	Infant mortality rate (0–1 year) per 1000 live births.	Infant mortality rate (0–1 year) is the probability of a child born in a specific year or period dying before reaching the age of one, if subject to age-specific mortality rates of that period. Infant mortality rate is strictly speaking not a rate, but a probability of death derived from a life table and expressed as rate per 1000 live births.	Ensure healthy lives and promote wellbeing for all at all ages.
Variable 5	Literacy rate of 15–24 year-olds, women and men.	The literacy rate of 15–24 year-olds is defined as the proportion of the population aged 15–24 years who can both read and write with understanding a short simple statement on everyday life. Literacy, in addition to the ability to read and write with understanding a short simple statement, generally also encompasses numeracy, that is, the ability to make simple arithmetic calculations.	Ensure that, by 2030, children everywhere, boys and girls alike, will be able to complete a full course of primary schooling.
Variable 6	Maternal mortality ratio per 100,000 live births.	The maternal mortality ratio (MMR) is the annual number of maternal deaths from any cause related to or aggravated by pregnancy or its management (excluding accidental or incidental causes) during pregnancy and childbirth or within 42 days of termination of pregnancy, irrespective of the duration and site of the pregnancy, per 100,000 live births, for a specified year.	Improve maternal health. Reduce, between 2015 and 2030, the maternal mortality ratio.
Variable 7	Attended by skilled health personnel, percentage.	The proportion of births attended by skilled health personnel is the proportion of total live births that are attended by a skilled birth attendant trained in providing life saving obstetric care.	Improve maternal health. Assistance by properly trained health personnel is key to lowering maternal deaths.
Variable 8	Children 1 year old immunized against measles, percentage.	The proportion of 1 year-old children immunized against measles is the proportion of children under one year of age who have received at least one dose of measles-containing vaccine. Children under one year of age who have received a measles vaccine are estimated as the percentage of children aged 12–23 months who received at least one dose of measles vaccine any time before the survey or before the age of 12 months.	Reduce child mortality. Reduce by two-thirds, between 2015 and 2030, the under-five mortality rate.
